# Virome Profiling of an *Amur leopard cat* Reveals Multiple Anelloviruses and a Bocaparvovirus

**DOI:** 10.3390/vetsci9110640

**Published:** 2022-11-17

**Authors:** Yang Liu, Lanshun Sun, Zhongzhong Tu, Sheng Sun, Yue Sun, Le Yi, Changchun Tu, Biao He

**Affiliations:** 1 Changchun Veterinary Research Institute, Chinese Academy of Agricultural Sciences, Changchun 130122, China; 2 Provincial Wildlife Disease Monitoring Station of Shuanghe, Heihe 164400, China; 3 Jiangsu Co-Innovation Center for Prevention and Control of Important Animal Infectious Diseases and Zoonosis, Yangzhou University, Yangzhou 225009, China

**Keywords:** wildlife, *Amur leopard cat*, viral metagenomics, anellovirus, parvovirus

## Abstract

**Simple Summary:**

Wildlife constitutes an essential part of the biodiversity on our planet. However, many factors put numerous species at the risk of extinction, among which infectious diseases are largely neglected. In this study, we report a pan-virome profiling of a rescue-failed Amur leopard cat. The results revealed abundant bacteriophages and multiple mammalian viruses, i.e., anelloviruses and a bocaparvovirus, in various organs of the animal, but no RNA viruses. Although the pathogenicity of anellovirus and bocaparvovirus is unknown, they showed very close phylogenetic relationships with other feline viruses, suggesting the circulation of certain viruses among domestic and wild felines. To our knowledge, this is the first report describing the virus diversity harbored by Amur leopard cats, which helps to understand the interaction of viruses between domestic animals and wildlife.

**Abstract:**

As a small top predator, Amur leopard cat (*Prionailurus bengalensis euptilurus*) is widely distributed in northeast Asia and plays an important role in the control of small rodent populations and in the maintenance of ecological equilibrium. However, the viruses harbored by this creature have been rarely investigated. Here, we report the DNA and RNA eukaryotic virome profiling of an injured Amur leopard cat followed by PCR validation, which revealed diverse anelloviruses in multiple organs and a bocaparvovirus in the lymph, but no RNA viruses. These anelloviruses have diverse genomic structures and are classified into four phylogroups with viruses of various felines, while the bocaparvovirus is extremely similar to those recovered from diarrheal domestic cats, illustrating the transmission of the virus between domestic animals and wildlife. These data provide the first insight into the genetic diversity of *Amur leopard cat* viruses, highlighting the need for further investigation of wild animals.

## 1. Introduction

Constituting a fundamental part of the biodiversity on our planet, wildlife provides the indispensable resource and natural ecosystem we depend on. However, it is facing increasing challenges such as climate change and human disturbance, resulting in that many species are at the edge of extinction due to the loss of the natural habitats [1]. In addition, a lot of wild animals are considered bushmeats or as having medical effects, attracting unscrupulous illegal hunting and overexploitation that further accelerate their extinction rate [2]. Therefore, wildlife conservation is of urgent interest to restore their populations as well as to protect the homeland of all creatures on the planet. Another threat to wildlife is infectious diseases that have long been ignored but have already led to catastrophic disasters for some species [3]. Due to the contagious caprine pleuropneumonia caused by *Mycoplasma capricolum* subsp. *Capripneumoniae*, the Tibetan antelope population in China’s Qinghai-Tibet Plateau shrunk ~16% in 2012 [4], while a fungus causing white-nose syndrome has almost wiped out the entire bat colony in some natural habitats of North America [5].

Notably, wildlife has been recognized as the natural reservoir of many zoonotic pathogens, and the spillover of wildlife viruses to human and/or domestic animals often results in deadly emerging infectious diseases (EIDs) [6], e.g., the bat-borne Marburg and Nipah virus lead to human fatal hemorrhagic fevers [7], and fox rabies viruses cause rabies outbreaks in livestock [8]. Therefore, the investigation and surveillance of wildlife viruses are essential not only for wildlife conservation but also for public health. 

Here, we report the eukaryotic virome profiling of a rescue-failed *Amur leopard cat* (*Prionailurus bengalensis euptilurus*), which reveals multiple anelloviruses and a bocaparvovirus. As a small top predator, the *Amur leopard cat* is listed as Least Concern by the International Union for Conservation of Nature and Natural Resources [9]. This species is widely distributed in northeast Asia and plays an important role in the control of small rodent populations and in the maintenance of ecological equilibrium. However, there is currently little knowledge of *Amur leopard cat* viruses. The data revealed here provide the first insight into the diversity of *Amur leopard cat* viruses.

## 2. Materials and Methods

### 2.1. Sample Collection and Species Identification

The injured *Amur leopard cat* was discovered by a local villager and transported to Shuanghe Wildlife Disease Monitoring Station for rescue. The animal did not survive after three days of medical treatment, and it was subjected to necropsy at the station. Its lung, brain, kidney, rectum, and mesenteric lymph nodes were collected and immediately cryo-transported to our laboratory, where they were stored at −80 °C. The species was morphologically identified by the staff at the station and further confirmed by sequencing the mitochondrial cytochrome coxidase subunit I gene (COI) [10].

### 2.2. Sample Pretreatment and High-Throughput Sequencing

All samples were individually subjected to virome profiling using our multiple displacement amplification (MDA)-based DNA and meta-transcriptomic (MTT)-based RNA viromic methods [11]. A small piece (~0.2 g) of each organ was cut and ground using 3 mL sterile phosphate-buffered saline solutions. After homogenization, samples were sequentially subjected to centrifugation at 12,000× *g* at 4 °C for 5 min, filtration through 0.45-µm-pore-size membranes (Millipore, Boston, MA, USA), and free nucleic acid digestion with a mixture of DNase I and RNase A (TaKaKa, Dalian, China). For the MDA method, viral DNA was extracted using a DNeasy Blood & Tissue kit (Qiagen, Hilden, Germany) and isothermally amplified using an Illustra GenomiPhi V2 DNA Amplification kit (GE, Fairfield, CT, USA) as per the manufacturer’s manual. The products were purified using a QIAquick PCR Purification Kit (Qiagen, Hilden, Germany) with one µg used to Illumina pair-end (150 bp) sequencing at an Illumina NovaSeq 6000 sequencer. For the MTT method, viral RNA was extracted using Trizol reagent (Invitrogen, Carlsbad, CA, USA) and subjected to rRNA depletion using the RiBo-Zero Magnetic Gold kit (Epicentra Biotechnologices, Madison, WI, USA). The remaining RNA was subjected to RNA-seq using an NEBNext Ultra directional RNA library prep kit (NEB, Ipswich, MA, USA).

### 2.3. Viral Metagenomic Analysis

The data processing and analyses were similar to our previous procedure [11]. Briefly, the initial quality control was performed using fastp version 0.20.0. High-quality reads were subjected to host genome removal using bowtie2 and rapid metagenomic assignment using kraken2 version 2.0.9b. Those unassigned reads of MDA and MTT libraries were mixed together, respectively, and de novo assembled using MEGAHIT version 1.1.3. Contigs of ≥1 kb were retained for BLASTx search with e-value cutoff of 1e-10 against our refined eukaryotic viral reference database (EVRD) version 1.0 aa branch for eukaryotic virome profiling, which allows us to avoid detecting false positive signals [12]. Besides, we also searched these contigs against the bacteriophage aa sequences of Genbank (division gbphg). All reads were mapped back to virus-like contigs using bowtie2 and their coverages and depths were counted using Samtools version 1.10.

### 2.4. PCR Validation

PCR was used to validate the viromic results and gap-filling. Primers were designed using Primer Premier5 (Supplementary Table S1). Viral DNA of each sample was extracted as described above. The PCR reaction was carried out using Phanta^®^ Max Super-Fidelity DNA Polymerase (Vazyme, Nanjing, China) with a thermocycler program of pre-denaturation at 95 °C for 3 min, 35 cycles of denaturation at 95 °C for 15 s, annealing at 59 °C for 15 s (or adjusted according to different primer pairs) and extension at 72 °C for 10 s, and a final extension at 72 °C for 5 min. The product was inspected by electrophoresis using 1% agarose gel with the expected one being directly sequenced on an ABI 3730xl DNA analyzer (Comatebio, Changchun, China).

### 2.5. Genomic Characterization and Phylogenetic Analyses

The putative open reading frame (ORF) was identified by NCBI ORF Finder (https://www.ncbi.nlm.nih.gov/orffinder (accessed on 26 July 2022)). The genomic structure was illustrated using SeqBuilder tool within DNAstar package version 7.4.0 (Lasergene, Madison, WI, USA). The genetic reference sequences were retrieved from Genbank and aligned with the viral sequences obtain in this study using Muscle module within the MEGA7 package. The maximum-likelihood phylogeny was inferred using MEGA7 with the best model assessed using the Akaike information criterion implemented in ModelFinder and assessed using the bootstrap analysis of 1000 replicates.

## 3. Results

### 3.1. Overview of the Virome

**Figure 1:**
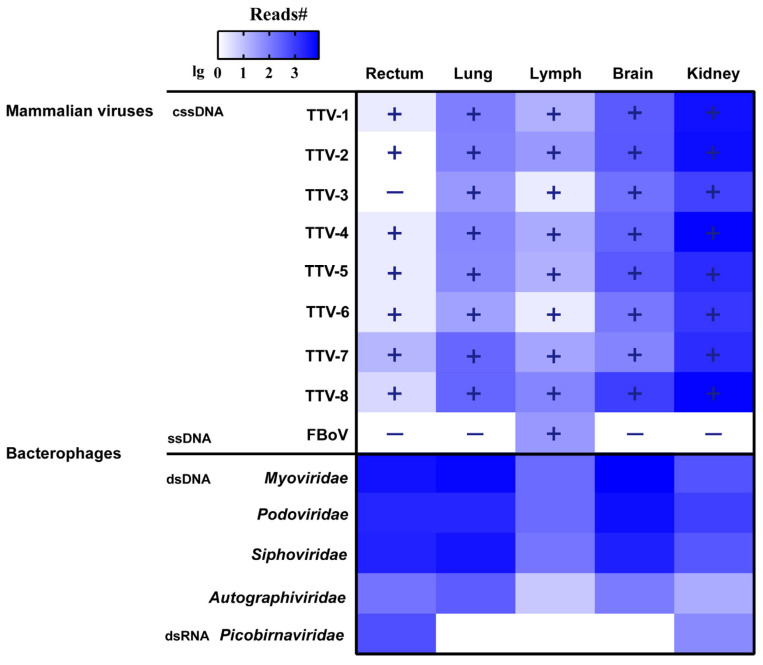
Overview of the virome profiling and the PCR detection of the *Amur leopard cat*. The heatmap shows the read numbers of these viruses in the five samples. The plus and minus symbols indicate the results of PCR detection. cssDNA: circular single-stranded DNA viruses; ssDNA: linear single-stranded DNA viruses; dsDNA: double-stranded DNA viruses; dsRNA: double-stranded RNA viruses; TTV: torque teno virus.

In January 2021, a female *Amur leopard cat* adult with a severe neck injury was rescued by the Provincial Wildlife Disease Monitoring Station of Shuanghe, Xunke, Heilongjiang Province, China. However, unfortunately, medical measures including neck fixation and fluid infusion failed to save its life in the following three days of treatment. Follow-up necropsy did not reveal any abnormal lesions in its internal organs, as well as any diarrhea, indicating that this animal was healthy and the severe injury on its neck caused its death. As a rare opportunity to investigate the natural baseline of viruses carried by this species, its lung, brain, kidney, rectum, and mesenteric lymph nodes were immediately collected for viral metagenomic examination. As a result, MDA and MTT generated 31.0 and 25.0 gigabase (Gb) of high-quality reads with 6.2 ± 1.0 and 5.0 ± 0.4 Gb per library, respectively. The virome profiling uncovered diverse and abundant bacteriophages in these samples, but with only a limited number of mammalian viruses (Figure 1). MDA captured abundant anellovirus reads in the kidney, brain, and lung samples, but few in the lymph and the rectum (Figure 1). This technique also detected a bocaparvovirus in the lymph. However, MTT did not detect any RNA mammalian viruses, though bacteria infecting double-stranded RNA picobirnaviruses were abundant in the rectum and the kidney (Figure 1). These anellovirus-like contigs were annotated to eight variants, i.e., torque teno virus (TTV) 1–8, within the species *Torque teno felid virus 1* of the genus *Etatorquevirus*, while all bocaparvovirus-like sequences derived from a single virus due to their similar identities to a common reference sequence. Follow-up validation with PCR methods targeting the nine viruses were highly consistent with the virome profiling. The PCR detection only identified bocaparvovirus in the lymph but not in other organs (Figure 1), while the eight TTVs were detected in almost all samples, except for TTV-3 negative in the gut. Of note is that both of virome and PCR detections showed the positivity of the eight TTVs in the brain, though it is widely recognized that the brain is free of virus under healthy conditions [13].

### 3.2. Genomic and Phylogenetic Characterization of Anelloviruses

**Figure 2:**
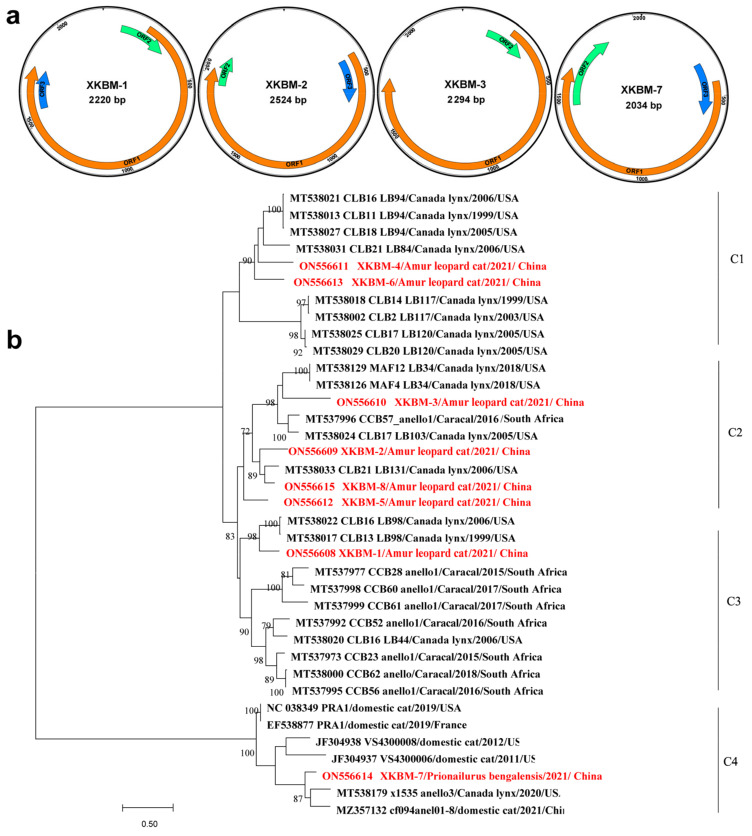
Genomic characterization of anelloviruses. (**a**) The genomic structures of four representative anelloviruses. The orange ORF represents ORF1; the green one, ORF2; the blue one, ORF3. (**b**) The phylogenetic analysis of ORF1 aa sequences. Viruses identified here are highlighted in red. Bootstrap values of ≥70 are shown next to nodes.

TTVs are a large group of single-stranded DNA viruses with a circular genome of 2.1–3.9 kb in length [14]. They are very rich in genetic diversity and are currently divided into 14 genera within the family *Anelloviridae* [15]. Frequently recovered by virome profiling of animals, TTVs have a wide host spectrum, but they are not directly associated to animal diseases [16]. We obtained the complete genomes of the eight TTVs (Genbank accession numbers: ON556608-ON556615) by de novo assembly. They were 2034–2524 nucleotides (nt) long with most encoding TTV-typical open reading frames (ORFs) 1, 2, and 3. These TTVs showed diverse genomic structures. For example, ORF3 was absent in TTV XKBM-3 and 4, and ORF2 overlapped at either the amino or carboxyl terminus of ORF1 (Figure 2a), which were also validated by PCR amplification and Sanger sequencing. The genetic reference sequences of the eight viruses were retrieved from Genbank, and their ORF1 sequences were aligned with the counterparts of the eight TTVs. The maximum-likelihood phylogeny was inferred using MEGA7 with the best model of rtREV+G+F and 1000 bootstrap replicates. As shown in Figure 2b, the eight viruses were genetically divergent from each other with 22.1–59.1% amino acid (aa) similarities and were classified into four clusters within the species *Torque teno felid virus 1* of genus *Etatorquevirus*. The eight viruses were 50.9–79.7% aa similar to their genetic neighbors, as are all viruses recovered from felines, such as lynxes (*Lynx canadensis* and *Lynx rufus*) from the USA, caracals (*Caracal caracal*) from South Africa, and domestic cats (*Felis catus*) from China. 

### 3.3. Genomic and Phylogenetic Characterization of Parvovirus 

**Figure 3:**
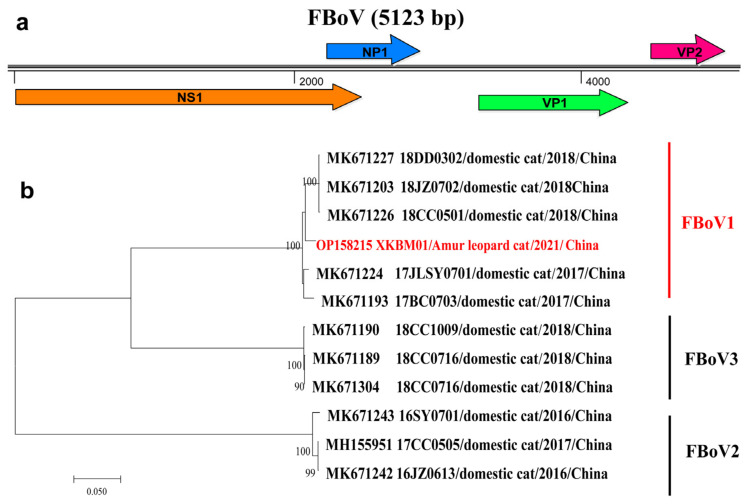
Genomic structure (**a**) and phylogenetic relationship (**b**) (based on NS1 gene) of FBoV XKBM01, which is highlighted in red in the phylogenetic tree. Bootstrap values of ≥70 are shown next to nodes.

Parvoviruses are a large group of single-stranded, linear DNA viruses with a ~5.0 kb long genome. Some parvoviruses are highly pathogenic to felines, e.g., feline parvovirus is responsible for the feline panleukopenia that is a contagious disease for cats with high mortality and morbidity [17]. Members within the genus *Bocaparvovirus* of the family *Parvoviridae* can cause respiratory and gastrointestinal diseases in humans and other animals [18]. Feline bocaparvoviruses (FBoV) were reported to cause hemorrhagic enteritis in cats [19]. Currently, FBoVs have been discovered across the world and are classified into three genotypes [20]. Here, the nearly complete genome (Genbank accession number: OP158215) of the bocaparvovirus (XKBM01) has been successfully obtained by de novo assembly and PCR-based gap-filling. It is 5123-nt long and of typical bocaparvovirus genomic structure with four encoding genes (Figure 3). The phylogenetic analysis based on the NS1 gene showed that XKBM01 fell into the cluster of FBoV1 with the closest genetic relationships (>97.6% nt similarity) with three cat BoVs (18CC0501, 18DD0302 and 18JZ0702), which were recovered from domestic cats (*Felis catus*) with diarrhea in Changchun, Dandong, and Jinzhou in northeast China.

## 4. Discussion

In this study, we conducted a pan-virome profiling of these multiple organs of an injured *Amur leopard cat* using a combination of MDA and MTT methods. The results revealed abundant bacteriophages, multiple anelloviruses, and a bocaparvovirus. It is not surprising that bacteriophages have high abundance in the virome since they infect a wide range of bacteria and dominate the animal virosphere [21]. MDA has a known bias to circular single-stranded DNA (cssDNA) viruses, but the modified version used here has a permissible capability to capture linear ssDNA and double-stranded DNA (dsDNA) viruses (Figure 1) [11]. Our previous evaluation also indicated that MTT is a powerful RNA virome technique with excellent sensitivity to detect all genome types of RNA viruses [11]. The abundant bacteriophages adequately proved that the two methods are capable of profiling diverse viruses (Figure 1). In addition, the necropsy showed very normal appearances of the internal organs, indicating that the cat did not contract any viral diseases. Altogether, that only a few DNA mammalian viruses were detected in the *Amur leopard cat* should not be ascribed to the preference of MDA and the incapability of MTT, but rather be explained by the status of limited viruses harbored by the animal at sampling. It is obvious that such data cannot represent the virus spectrum of this mammalian species as it was only obtained from a single animal; more diverse viruses will undoubtedly be discovered if more individuals are investigated in the future. 

The identification of multiple TTVs in the brain by both MDA and PCR detections is interesting. Previous studies also detected TTVs in the brain samples of pigs [22] and in the cerebrospinal fluids of humans [23]. TTVs constitute a common part of animal viromes with wide tissue/organ tropisms and are often present in the viromes of the gut and lung, especially with high virus load in blood [24]. Here, we cannot determine the physiological or pathological route of TTV to enter the brain, but there exists one possibility for the presence of TTV in the leopard cat brain. Our precaution can have prevented the nucleic acid template contamination in MDA treatment and PCR detection, but the brain tissue could have been contaminated by the TTV-containing blood/sample when the skull was opened for sampling, which could also have resulted in the presence of bacteriophages in the brain. 

Of note is that the anelloviruses and bocaparvovirus are all related to the viruses of other feline species. Particularly, the bocaparvovirus is as high as 97.6% nt similar to the three BoVs causing diarrhea in domestic cats from a broad area of northeast China, suggesting that the virus could transmit between *Amur leopard cats* and domestic cats. This is a concerning phenomenon, that some viruses can circulate between domestic animals and wildlife, which poses a great challenge to control and prevent some major animal diseases. For example, rabies virus is very difficult to eliminate from China due to its circulation between dogs and wild carnivores, and often cause rabies outbreaks in domestic livestock [25]. In addition, African swine fever viruses (ASFV) emerged originally in domestic pigs and has delivered a catastrophic disaster to the pig industry in China, and the spillover of ASFV into wild boars poses a severe threat to the animals, which also makes it very hard to eradicate it in China [26].

In summary, EIDs pose a substantial challenge to wildlife conservation. To tackle this issue, many measures and policies should be comprehensively considered and implemented, e.g., the practice of house feeding can cut the transmission of viruses between domestic animals and wildlife, and the vaccination of wildlife can effectively prevent its exposure to those deadly viruses. Furthermore, investigation and surveillance of the virus diversity harbored by wildlife should be intensified, which not only helps to formulate and implement measures to control and prevent EIDs but is also necessary for disease diagnosis and prediction in wildlife conservation.

## Data Availability

The complete genomes of the eight TTVs and the one FBoV were deposited in Genbank under accession numbers ON556608-ON556615 and OP158215. The MDA and MTT raw data were available in the NCBI Sequence Read Archive (SRA) under accession numbers PRJNA847403.

## Supplementary Materials

The following supporting information can be downloaded at: https://www.mdpi.com/article/10.3390/vetsci9110640/s1, Table S1: Primer information used in this study.
