# Supplementary material for: Virome Profiling of an Amur leopard cat Reveals Multiple Anelloviruses and a Bocaparvovirus
Source: Vet Sci. 2022 Nov 17;9(11):640. doi: 10.3390/vetsci9110640 (PMC9693195; doi:10.3390/vetsci9110640)
Supplement: Supplementary file 1 [file vetsci-09-00640-s001.zip › vetsci-2021193-SI.pdf]

Supplementary Table S1. Primer information used in this study.

Application	Primer name	Sequence (5'-3')	Target
Species identification	COI-TF	5` GGTCAACAAATCATAAAGATATTGG 3`	
	COI-TR	5`TAAACTTCAGGGTGACCAAAAAATCA 3`	COI
PCR detection	ACF1	5`ATCTTGAGCGTGAGCAGCTT3`	
	ACR1	5`AATTAGGGAGCCGCTGTGTC 3`	TTV-1
	ACF2	5` CTTCAATCAGGTCCGCTGGT 3 `	
	ACR2	5` TGACGAAACAGGGTCCGAAG 3`	TTV-2
	ACF3	5` GTACTCGCCTCTAGTAGATA 3`	
	ACR3	5` AACCAGTCTCACAACTTAGC 3`	TTV-3
	ACF4	5` GACCTCAATAGAACTGGAC 3`	
	ACR4	5` GGTAAGGAGTCCAGTTCTA 3`	TTV-4
	ACF5	5` TTCCGACTCCTCTTCACTT 3`	
	ACR5	5` CAAAAACCCCAACACCATC 3`	TTV-5
	ACF6	5` GACACGGACGGGGAAGAAG 3`	
	ACR6	5` CAGCAGTCAAGTGAAAGTT 3`	TTV-6
	ACF7	5` GCCGTCACGAGAGAAGTTT 3`	
	ACR7	5` TGGGGACAGCCAGGAGTAT 3`	TTV-7
	ACF8	5` GTCTTAGGCTTTGTTGCTT 3`	
	ACR8	5` TCCTGGTCTGATGACTGTT 3`	TTV-8
	BCF	5` TCTCTGGTTGGCTTTCATA 3`	
	BCR	5' GTAGCTCGTACCTGGTGTA 3'	FBoV
PCR-based gap-filling	P1XF	5` TCAGTCTCACATGTACATGAA 3`	
	P2SR	5` AATGTGTACACCCGCTGTAA 3`	
	P2XF	5` TTGACACTAGACGTTATTATCA 3`	
	P3SR	5` CGAGAGAGACAGATGGATAG 3`	
	P4SF	5` TTGACGAGAGTATGGACCT 3`	
	P4XF	5` CTATGCTGGATTCGGCTGA 3`	FBoV
